# Supplementary material for: You can’t teach speed: sprinters falsify the deliberate practice model of expertise
Source: PeerJ. 2014 Jun 26;2:e445. doi: 10.7717/peerj.445 (PMC4081292; doi:10.7717/peerj.445)
Supplement: Tables S1--S3 [file peerj-02-445-s001.docx]

**Supporting Tables**

**Supporting Table 1.** Quotes from peer-reviewed articles illustrating the Deliberate Practice Model (DPM) of expertise.

“Many characteristics once believed to reflect innate talent are actually the result of intense practice *extended for a minimum of 10 years*.” [1; p. 363, emphasis added].

“We have shown that expert performance is acquired slowly over a very long time as a result of practice and the highest levels of performance and achievement appear to require at least around 10 years of intense prior preparation.” [1; p. 366, emphasis added].

“If only well-established domains with a large number of active individuals are considered we know of only a small number of exceptions to the *general rule* that individuals require *10 or more years* of preparation to attain international-level performance.” [1; p. 366, emphasis added].

“Our framework made two important predictions…First, the past amounts of deliberate practice is directly related to the individual’s current performance. More specifically, *expert performance is not reached with less than 10 years of deliberate practice.*” [1; p. 372, emphasis added].

“…the past amount of deliberate practice is directly related to the individual’s current performance. More specifically, *expert performance is not reached with less than 10 years of deliberate practice*.” [1; p. 372, emphasis added].

“Because of the high costs to the individuals and their environments of engaging in high levels of deliberate practice and the overlap in characteristics of deliberate practice and other known effect training situations, one can infer that high levels of deliberate practice are necessary to attain expert level performance. Our theoretical framework can also provide a sufficient account of the major facts about the nature and scarcity of exceptional performance. Our account does not depend on scarcity of innate ability (talent) and hence agrees better with the earlier reviewed findings of poor predictability of final performance by ability tests.

We attribute the dramatic differences in performance between experts and amateurs-novices to similarly large differences in recorded amounts of deliberate practice.” [1; p. 392].

“*No case* has been encountered of anyone reaching the highest levels of achievement in chess-playing, mathematics, music, or sports without devoting thousands of hours to serious training” [2; p. 406, emphasis added].

“… it is possible to account for the development of elite performance among healthy children without recourse to unique talent (genetic endowment)- excepting the innate determinants of body size.” [3; p. 4].

“…distinctive characteristics of elite performers are adaptations to extended and intense practice activities that selectively activate dormant genes that all healthy children’s DNA contain…” [3; p. 4].

“Consistently and overwhelmingly, the evidence showed that *experts are always made not born.*” [4; p. 1, emphasis in original].

“Our research shows that *even the most gifted performers need a minimum of ten years (or 10,000 hours)* of intense training before they win international competitions.” [4; p. 4, emphasis added].

“It appears that the genes necessary to attain such adaptations and expert skills can be activated in healthy children – the only clear exceptions to date being genes that control body size and height.” [5; p. 346].

“Finally, there is *no evidence* that international competitions can be won, even by the most gifted, with less than a decade of prior intense involvement in domain related activities…” [5; p. 348, emphasis added].

“Although several factors influence level of achievement, there appears little doubt that extensive experience in a sport is necessary to reach the very highest levels of performance” [6; p. 5].

**References**

1. Ericsson KA, Krampe RT, Tesch-Römer C (1993) The role of deliberate practice in the acquisition of expert performance. Psychological Review 100: 363-406.

2. Howe MJ, Davidson JW, Sloboda JA (1998) Innate talents: reality or myth? Behav Brain Sci 21: 399-442.

3. Ericsson KA (2007) Deliberate practice and the modifiability of body and mind: toward a science of the structure and acquisition of expert and elite performance. International Journal of Sport Psychology 38: 4-34.

4. Ericsson KA, Prietula MJ, Cokely ET (2007) The making of an expert. Harvard Business Review July-August: 1-8.

5. Ericsson KA, Ward P (2007) Capturing the naturally occurring superior performance of experts in the laboratory: towards a science of expert and exceptional performance. Current Directions in Psychological Science 16: 346-350.

6. Williams MA, Ford PR (2008) Expertise and expert performance in sport. International Review of Sport and Exercise Psychology 1: 4-18.

**Supporting Table 2.** Quotes from published autobiographies and biographies of male and female 100 m and 200 m Olympic gold medal winners. Athletes are listed alphabetically.

**Evelyn Ashford (b. 1957) (1984: 100 m)**

Hornbuckle AR, (1995) Evelyn Ashford Washington. In: Porter DL, editor. African-American sports greats: a biographical dictionary. Westport, CT: Greenwood Press. pp. 361-363.

“The University of California at Los Angeles (UCLA) awarded Evelyn one of the first athletic scholarships given to a woman. [she] first gained prominence as a UCLA freshman in 1976 [age 19]. After finishing second in the 1976 Association of Intercollegiate Athletics for Women (AIAW) 100-meter dash, she made the U. S. Olympic team and finished fifth at the Montreal, Canada Summer Olympic Games.” p. 361.

Davis MD (1992) Black American women in Olympic track and field. Jefferson, NC: McFarland & Company, Inc., Publishers.

“…the 19-year-old sprinter, virtually unknown, finished a surprising fifth in the 100 meters.” p. 9.

“Evelyn ran competitively for the first time when she was a high school senior [1975]. Her math teacher was impressed with Evelyn’s athletic skills and suggested she participate in track. At the local high school she joined the boys’ track team because there wasn’t one for girls. She beat the school’s star football player every time she ran against him in the 50-yard dash. In high school Evelyn gained regional and statewide reputations, and UCLA gave her a track scholarship in 1975…In the summer of 1976 she went to Montreal as a member of the U. S. team.” p. 12.

**Usain Bolt (b. 1986) (2008: 100 m, 200 m; 2012: 100 m, 200 m)**

Bolt U (2010) Usain Bolt: 9.58: my story being the fastest man on Earth. London, UK: HarperSport.

“I chose to be a sprinter, not only because I was the fastest kid in school…” p. 13.

“Much as I loved football and cricket, running came so easy for me…I was the fastest, not only in school, but in the whole parish of Trelawny. After a few years in high school when I put my mind to training, I was the quickest junior in Jamaica, then the world.” p. 14.

“As a child I was good at all sports, especially running, cricket and football.” p. 28.

“I didn’t really start trying until grade nine, when I was about 14, … 2002, I was winning the World Junior Championships. Training had helped, but I also realised I had talent, real raw talent.” p. 34.

Glen Mills, Bolt’s coach noted “…he is a fantastic athlete – once in a lifetime you see this kind of talent.” p. 101.

Lorna Thorpe, head of sports, William Knibb High School recalled, “He wanted to run every race in school for his house team. He did 100, 200, 400, 1500, the relays and even cross country, but didn’t take training very seriously at first…We told him he had a gold mine in his legs.”

Wellesley (Gideon) Bolt, Usain’s father recalled that when Usain was in high school, “It annoyed me, though, when he didn’t dedicate himself to training…Even when we knew that he had a special talent as an athelet…” p. 281.

Cantor G (2011) Usain Bolt. Detroit, MI: Lucent Books.

“When he entered William Knibb Memorial High School, however, even his cricket coach could see that it was the wrong game for him. ‘I’d look at that stopwatch as he ran up to bowl and think, ‘There’s something wrong with this watch,’ said coach Dwight Barnett. ‘No kid can run that quickly.’” p. 16.

“…Pablo McNeil, who had competed in the one-hundred-meter dash in 1964 and 1968 Olympics...recalled his early efforts to make Usain a sprinter. ‘He was so stubborn…But you didn’t have to be a prophet to know he was going to break world records.” p. 16.

Bolt became a professional sprinter in 2004 at the age of 18 and ran 200 m in under 20 seconds at the CARIFTA Games shattering the world junior record for the event. He was the first teenager to run 200 m in less than 20 s (19.93 s). He appeared at the Olympic Games in Athens the same year but was injured and did not advance past the first round in the event. pp. 24-25.

Irving R (2010) Usain Bolt: phenomenal track athlete. In: Irving R, Charlton V, editors. Jamaican gold: Jamaican sprinters. Kingston, Jamaica: University of the West Indies Press. pp. 54-59.

“By age twelve years, he was the fastest boy in his school…by puberty his speed on the pitch was remarkable….He entered William Knibb Memorial High School at about thirteen years of age [1999] and continued focusing on cricket. The cricket coach noticed is extraordinary speed on the pitch and recommended that he try track and field events…Usain won his first high school championship medal in 2001, placing second in the 200 metres in 22.04 seconds. However, his first appearance on the world stage at the 2001 IAAF World Youth Championship was disappointing…” p. 54

**Gail Devers (b.1966) (1992: 100 m. 1996: 100 m)**

Hornbuckle AR (1995) Gail Devers. In: Porter DL, editor. African-American sports greats: a biographical dictionary. Westport, CT: Greenwood Press. pp. 69-72.

“Devers, at age 15 began track and field as a middle-distance runner at National City’s Sweetwater High School, south of San Diego…By 1987 (age 21), Gail had achieved world class times of 10.98 seconds in the 100 meters and 22.71 seconds in the 200 meters.” p. 70

**Florence Griffith-Joyner (1959-1998) (1988: 100 m, 200 m)**

Hornbuckle AR (1995) Florence Griffith Joyner. In: Porter DL, editor. African-American sports greats: a biographical dictionary. Westport, CT: Greenwood Press. pp. 125-127.

“Griffith, who discovered her running ability “chasing jack rabbits” at her father’s home in the Mojave Desert began competing at age 7 in track and field meets …She remembered “always winning” the 50- and 70-meter dashes throughout elementary and junior high school.” p. 125.

“As a senior at David Jordan High School in 1978, she established school records of 10.86 seconds for 100 yards and 24.4 seconds for 220 yards.” p. 126.

Davis MD (1992) Black American women in Olympic track and field. Jefferson, NC: McFarland & Company, Inc., Publishers.

“At 14 she won the annual Jesse Owens National Youth Games…she won the same meet the following year…” p. 65.

“She graduated from Los Angeles’ Jordan High School in 1978; she had set school records in sprinting and the long jump.” p. 66.

**Bob Hayes (1942-2002) (1964: 100 m)**

Hayes B (1990) Run, bullet, run: the rise, fall, and recovery of Bob Hayes. New York, NY: Harper & Row.

“For all intents and purposes, I spent only about two years running track seriously. If I ignore my minor-league track experience in high school, when I was playing baseball at the same time, my entire track career consisted of spring 1961; spring and summer 1962 and 1963; and spring and early fall 1964, the year I missed the whole summer season with my hamstring injury.” p. 36.

“I never lifted weights when I was running.” p. 36.

“While I was at the Olympics, it never crossed my mind that I had peaked at age twenty-one.” p. 37.

“When I was eleven I got into my first organized sports, Little League baseball...I could really get the ball out in center field.” p. 41.

“I had run track in seventh and eighth grades, and in a span of two weeks, I entered the 50-, 100-, 200-yard dashes; the 400- and 880-yard relays; and the high jump, long jump, and pole vault, and won them all.” p. 42.

“He [Bill Logan, a friend of Bob Hayes’s brother, Ernest Hayes] knew that I could run fast – he was first one who detected that I was real fast – and he always bet on me. When I was in ninth grade, he bet on me against a twelfth grader, whom I beat.” p. 42.

“…I had decided not to play anything except football when I was in eleventh grade. But in the spring of that year, I was fooling around in gym class while we were running outside on the blacktop road. The track team was practicing in a field next to the road, and I found out who their fastest sprinter was. I got next to him and told him, “I can beat you.” … And I beat him easily, by about five yards. Frank Cannon, the track coach, came running over and said, “I have to have you on my team. You have 9.6 speed. I’ve never had a sprinter that fast before.” p. 45.

“…they were afraid to report my time as 9.4 because nobody would have believed that a sixteen-year-old with hardly any experience or training had come within one-tenth of a second of the world record.” p. 46.

**Ben Johnson (b. 1962) (1988: 100 m, but disqualified for using Performance Enhancing Drugs (PEDs))**

Christie JR (1988) Ben Johnson: the fastest man on Earth. Toronto, Canada: McClelland-Bantam, Inc.

“The moment came in 1977. Ben was still a 93-pound weakling, and he looked younger than his fifteen years, but at school gym classes he was showing his natural ability to get out in front during runs. One day a group of buddies challenged him to a dash. “They were always on me, because I called myself fast but never showed it. One day in gym class, we were all properly warmed up and I knew it was time to do the job. We were all lined up. I looked across at the fastest guy and said, ‘Okay, you ready to race?”…Ben won the 100-metre race. His time – running in flat shoes because he didn’t own track spikes – was 11.8 seconds.” p. 53.

“Charlie [Francis] told Ben that he had some potential. ‘I could see that after his first few practices. But he had to stick with it to be any good.’” P. 59.

“In 1979 Johnson was the Canadian junior champion in 10.06 seconds…In 1980, at age 18, he was named to the drydocked Olympic team.” P. 66.

**Michael Johnson (b. 1967) (1996: 200 m)**

Johnson M (1996) Slaying the dragon. New York, NY: Regan Books.

“I remember my first real race. I must have been ten years old and I believe we were running a 50-yard dash…I remember running and realizing that I was moving faster than the other kids, that I was out in front, that I was winning. I remember the exhilaration that came from knowing that I was the FASTEST! … I didn’t even run track my freshman year in high school…It wasn’t until my junior year that I realized what I could accomplish as “A Runner.” pp. 3-4.

**Marion Jones (b. 1975) (2000: 100 m)**

Gutman W (2000) Marion Jones: the fastest woman in the world. New York, NY: Pocket Books.

“Even before Marion Jones entered junior high school there were flashes of athletic brilliance.” p. 13

“…in a gymnastics class…Within a month, the young Marion had a skill level equal to most of the older girls who had been practicing for years.” p. 13

“Pinecrest Junior High… Marion began to excel at both track and basketball. By 1989, when she was in eighth grade, she ran bests of 12.01 for 100 meters, 24.06 for 200 meters, and 56.73 for 400 meters. The times were incredible since Marion was just thirteen years old.” p. 14

In ninth grade “… she also won both 100- and 200-meter championships at the California High School State Meet.” pp. 16-17.

“Her sophomore year…she… was named High School Athlete of the Year. What’s more, here personal best times…were now world class times. At the end of the season the Track & Field News ranked her the tenth best in the country in the 100 and fifth best in the 200. All that at fifteen years of age.” p. 17.

“At age fifteen, she was already a world class runner and very close to being among the best.” p. 18.

Jones M (2004) Life in the fast lane. New York, NY: Time Warner Books

“And my speed? Well, I’ve been blessed. It has always been there.” p. 39.

“In 1989….Until then I had only been running against other high school kids and had no doubt that I was faster than everybody else. But in my sophomore year at Rio Mesa, I got my first chance to compete against elite runners. I was asked to an annual meet at UCLA… a very prestigious event…one that they don’t normally open to 14-year-old girls.” p. 40.

“Later in 1991, I was named National High School Athlete of the Year for the first of three straight years. I also qualified for the U.S.A. Track and Field Championships…When I placed sixth in the 100 m and fourth in the 200 m, I knew I’d be running alongside them [Evelyn Ashford and Gwen Torrence] soon.” p. 42.

“I’d started receiving letters from colleges at the beginning of my freshman year (by the end of my senior year I’d gotten more than 400 of them) and the vast majority wanted me for track.” p. 45.

“On the track, my junior year culminated in my participation in the 1992 Olympic trials. I did okay, finishing fourth in the 200 m and missing a spot on the U. S. squad for the Barcelona Games by only .07 seconds. In the 4x100 m relay, I was offered a place on the team as an alternate, but I turned it down without hesitation.” p. 47.

“I won the state title in the 100 m and 200 m for three straight years – so often, in fact, that by my senior year, I was hungry for a new challenge…I decided on the long jump…I knew I could jump – by then I could dunk a basketball – and of course I could run…I gave it a try at a meet, I jumped clear over the pit…a distance of more than 21 feet. I went to the state meet, jumped more than 22 feet and won the title, almost breaking the national high school long jump record. pp. 47-48.

Jones went to University of North Carolina to play basketball but she also competed in track. Two weeks after the end of the basketball season during her freshman year she was back on the track. Six weeks after the end of the basketball season, she was competing in the NCAA Division I Track and Field championships in Boise, ID in 1994. She placed second in the long jump, earning All-American status, sixth in the 200 m, but did not make the finals in the 100 m. “Given my extremely limited training, I was fairly content with these smaller victories, yet something in me still expected my performances to be at the highest level, even if I wasn’t training like the elite sprinter I aspired to be.” p. 61.

**Carl Lewis (b. 1961) (1984: 100 m, 200 m. 1988: 100 m)**

Lewis C, Marx J (1990) Inside track: my professional life in amateur track and field. Toronto: Fireside Book.

“Finally, I won a local Jesse Owens meet in Philadelphia, placing first in a 1973 long-jump competition for twelve-year-olds” p. 22.

“My time in the 100-yard dash kept getting better. First 9.7 seconds, then 9.6, and the times kept coming down. At the national age-group meet in Memphis, I ran the 100 in 9.3 seconds. In a year, my time had dropped from 10.6 to 9.3.” p. 28.

**Jesse Owens (1913-1980) (1936: 100 m, 200 m)**

Baker WJ (1986) Jesse Owens: an American life. New York, NY: Free Press.

Speaking of Owens’s coach, Charles Riley, “He certainly latched onto the young Owens, recognizing his natural athletic talent as a rough gem worth polishing….Riley’s investment soon began to pay dividends. About a year after he began his paternalistic training of Jesse, he timed him in the 100-yard dash at 11 seconds. …Then in 1928 Owens set his first two of innumerable records: 6 feet in the high jump, and 22 feet, 11¾ inches in the long jump. Both were new world marks for junior high athletes.” p. 22.

Owens failed to win a place on the 1932 Olympic team but “Shortly after the Los Angeles Games, several European athletes on their return home stopped over in Cleveland for an afternoon track meet against some American Olympians and the best local talent. Although none of the better foreign sprinters competed, young Owens salved his pride by winning the 100-meter and 200-meter races. In the long jump he finished second to Edward Gordon, the American Olympic gold medalist.” p. 29.

In 1933, during his senior year in high school, “From one meet after another he emerged without a single loss. In the final account, he finished first in 75 of the 79 races he ran in high school.” p. 31.

In mid-June 1933 Owens capped his remarkable high school career at the National Interscholastic Championship meet at Stagg Field, Chicago…In the 220-yard dash he set a new world mark of 20.7 seconds, and in the 100-yard dash his 9.4 seconds tied the world record.” p. 32.

**Wilma Rudolph (1940-1994) (1956, 4x100 m Bronze; 1960, 100 m, 200 m)**

Davis MD (1992) Black American women in Olympic track and field. Jefferson, NC: McFarland & Company, Inc., Publishers.

“… she earned a place on the 1956 U.S. women’s Olympic team…the scared 15-year-old…was going to her first Olympic Games…” p. 113.

Mallon W (1995) Wilma Rudolph. In: Porter DL, editor. African-American sports greats: a biographical dictionary. Westport, CT: Greenwood Press. pp. 288-291.

“She also excelled as a sprinter…During her junior year in high school, Rudolph qualified for the U.S. Olympic team in the 200 meters and 4x100 m relay. At the 1956 Melbourne, Australia Summer Olympic Games, Wilma helped the 400-meter relay team win a Bronze Medal in her first international appearance.” p. 289.

Rudolph W (1977) Wilma. New York, NY: New American Library.

After eighth grade basketball season, Rudolph’s basketball coach, Clinton Gray, started a girls track team. After couple of days of just jogging in practice, Gray lined up all his athletes on a curb while he stood in the middle of a field and asked them to run as fast as they could past him. “The first time I ran in the pack, I noticed that I picked up speed halfway there and I beat everybody else past the coach. Then this continued every day, and every day I’d be the first one past him.” p. 46.

After the ninth grade basketball season, Rudolph ran the 50, 75, 100, and 200 meters, on the relay team in meets around Tennessee. “I was thirteen years old, a ninth-grader, and believe it or not, I won every race I ran in that season.” p. 49.

“Right after Philadelphia [AAU Junior Championships], and just before my junior year in high school, Coach Temple and I had a long talk; it was the first time he talked to me about the Olympic Games. He said, “Wilma, I think that you have a chance to run in the Olympics, and I think that you should give it a try.” p. 80.

Schraff A (2004) Wilma Rudolph. The greatest woman sprinter in history. Berkeley Hts., NJ: Enslow Publishers, Inc.

“Sixteen-year-old Wilma Rudolph, a lanky girl from Tennessee, was taking part in her first Olympics. ….Only four years earlier, Wilma was wearing a heavy steel leg brace and orthopedic shoes.” p. 7.

“But in the championship game, Ed Temple saw a Wilma Rudolph he had not seen before. ‘The first time I set eyes on Wilma was when she was playing basketball,’ he said. But suddenly he was seeing her as a possible champion in another sport: Looking at Wilma, he saw a natural sprinter.” pp. 25-26.

“Though still a high school junior, Wilma Rudolph had gone to the Olympic Games in Melbourne, Australia, and won a bronze medal in the 400-meter relay race.” p. 32.

**Tommie Smith (b. 1944) (1968: 200 m)**

Smith T, Steele D (2007) Silent gesture: the autobiography of Tommie Smith. Philadelphia, PA: Temple University Press.

In fourth grade “…I was one of the best athletes in my grade then, I would only show it off in the schoolyard, or around my family.” p. 61.

“Eventually, I was able to do things most grammar school kids couldn’t do. As I entered eighth grade, I was long-jumping 17 or 18 feet, and I could come close to dunking a basketball.” p. 63.

“… and in basketball, where I could outjump everyone and outrun everyone on the fast break and where I was the school’s all-time leading scorer when I left, and for a long time afterward. But it was on the track that I excelled. In the typical meet, I would compete in all the jumps, run everything from the 100 to the 400 and all the relays…In my junior year, I broke a meet long-jump record by going 24 feet 2 inches, and I rarely even practiced.” p. 69.

“…one day in my junior year…Coach Burton told me to run it [a 440] during a meet…I told him [his coach] that I had never run the 440…I finished…and everyone was congratulating me. I’d run a 47.7, the best in the nation that year. By my senior year I was running it in 47.3, nearly a half second faster.” p. 69.

**Helen Stephens (1918-1994) (1936: 100 m)**

Hanson SK (2004) The life of Helen Stephens: the Fulton flash. Carbondale, IL: Southern Illinois University Press.

In her freshman year of high school “…she was stronger, leaner, more muscular, and faster on her feet than any kid they had seen before.” p. 20.

“Boys ran fifty yards in seven seconds flat to qualify for an F [high school athletic “letter”]. Helen thought she would like to try for a track letter, just to see. It was an odd goal for a girl, and an improbable one, even if she had the speed for it.” p. 21.

In spring 1933, Helen ran a 50-yd dash and she ran it in 5.8 seconds “Moore was skeptical, having clocked her in 5.8 seconds. When she ran it that fast a second time, he knew he had something….it was an incredible moment…to watch an untrained sophomore match the speed of champion girl sprinter Elizabeth “Betty” Robinson. Robinson held the outdoor 50-yard world record at 5.8….”

Her coach W. Burton Moore said “She’s Amazonian, outrunning my boys even when she gives’em a 100-yard lead in a 1 mile race.” p. 23.

**Gwen Torrence (b. 1965) (1992: 200 m)**

Hornbuckle AR (1995) Gwen Torrence. In: Porter DL, editor. African-American sports greats: a biographical dictionary. Westport, CT: Greenwood Press. pp. 358-361.

“At Columbia High School in Decatur, Gwen discovered her penchant for running as a tenth grader. The first time I saw Gwen run,” recounts Ray Bonner, a Columbia physical education teacher and football and track and field coach, “a football player by the name of Fred Lane lit out after her on the track. Fred couldn’t catch her. And he was fast. Bonner persuaded a reluctant Torrence to join the track and field team, stressing “that God gave her a gift, and if she didn’t use it, he was going to be very upset…She captured the 100 meters and 200 meters in the Georgia State championship and the Track Athletic Congress (TAC) Junior championship as a senior…” p. 359.

**Wyomia Tyus (b. 1945) (1964: 100 m. 1968: 100 m)**

Carlson LH (1995) Wyomia Tyus-Tillman. In: Porter DL, editor. African-American sports greats: a biographical dictionary. Westport, CT: Greenwood Press. pp. 349-351.

“Tyus set her first world record at the 1964 Tokyo Games..” [at age 21] p. 349.

“Tyus excelled in both basketball and track and field in high school. In 1962, Wyomia won the 100-yard dash at the Girl’s AAU championship in Los Angeles, California. She set an American age-group record of 11 seconds and lowered it to 10.9 seconds the following year.” p. 350.

Tyus W (2010) Wyomia Tyus. In: Walter JC, Tida M, editors. Better than the best: black athletes speak, 1920-2007. Seattle, WA: University of Washington Press. pp. 133-149.

“I started athletics in the seventh grade…they would always put me where I would go out for the pass, catch it, and out sprint everybody. I would even out sprint my brother.” p. 135.

“In junior high school…My training was playing ball with my brothers, but I could beat all the girls anyway…We would have track meets…I would go out there and I would run and I would beat them.” p. 136.

“In the summer of 1963, before I went to college, I went to Europe with the AAU team.” p. 138

“We went to the Olympic trials at Randall’s Island in New York in 1964, when I was eighteen.” p. 140.

Davis MD (1992) Black American women in Olympic track and field. Jefferson, NC: McFarland & Company, Inc., Publishers.

“…like several other female track stars, started athletic life as a high school basketball player but quickly changed to track when her athletic teachers noticed her speed. Wyomia Tyus is truly one of the most remarkable athletes ever to represent the United States. Before she was 18, she was touring Europe demonstrating her talent; before her 19^th^ birthday, she was setting world records.” p. 143.

**Supporting Table 3.** Two sprinters whose biographies contradict the DPM.

David Sime (b. 1936): Sime was a champion sprinter in the late 1950s. Sime’s ascent to world class status was meteoric. Sime attended Duke University on a baseball scholarship. After seeing Sime at a baseball game, the Duke track coach asked him if he would be like to try sprinting. He ran his first official 100 yd race in 9.6 s, 0.3 s slower than the world record at the time. Two months prior to his 20^th^ birthday, and after sprint training for just over one year [1], he broke the world record in the 220 yd low hurdles and the 220 yd dash [2]. An injury prevented Sime from competing in the 1956 Olympics, but he won the silver medal in the 100 m sprint at the 1960 Rome Olympics. During his brief career, Sime held world records at 100 yd, 220 yd, and the 220 yd low hurdles [2]. In summary, Sime became a world class sprinter with less than one year of deliberate practice.

Delano Meriwether (b. 1943): Meriwether was a champion sprinter in the early 1970s. Meriwether did not compete in track or other sports in high school [3] and took up sprinting while a practicing physician at the age of 27 in February 1970 after attending a local track meet [4]. Meriwether began training on local tracks, running up and down the stairs in his apartment building, and running in local track meets [4] without formal coaching [3]. He reported to have remarked to his wife, “Hey, I can beat those guys” while watching on television the USA track team competing in Europe during the summer of 1970. Later in 1970, Meriwether ran a 9.4 s 100yd dash, just 0.3 s off the world record at that time. In early 1971, he beat nine world class sprinters in a 60 yd dash at the National Invitational Meet in College Park, MD running the distance in 6.0 s, just 0.1 s slower than the world record for that distance. In 1971, he won the 100 yd dash at the U. S. Track and Field Championships tying the world record in a wind-aided 9.0 s. Meriwether’s athletic career was cut short by injuries, but he was the U. S. Indoor 60 yd dash champion in 1972, ran for several years as a master’s athlete setting the 200 m record of 20.8 s for the 35-39 age group in 1978 (www.usatf.org/statistics/records/). In summary, Meriwether went from “armchair” athlete to world class sprinter with less than one year of deliberate practice.

**References**

1. Terrell R (1956) A classic duel of speed. Sports Illustrated 4: 52-54.

2. Lawson G (1997) World record breakers in track & field athletics. Champaign, IL: Human Kinetics.

3. Treadwell S (1971) Hey, I can beat those guys. Sports Illustrated 34: 14-15.

4. Boyle RH (1971) Champion of the armchair athletes. Sports Illustrated 34: 20-25.
