# Supplementary material for: You can’t teach speed: sprinters falsify the deliberate practice model of expertise
Source: PeerJ. 2014 Jun 26;2:e445. doi: 10.7717/peerj.445 (PMC4081292; doi:10.7717/peerj.445)
Supplement: Figure S1 [file peerj-02-445-s002.docx]

**Supplemental Figure Legend**

Supplemental Figure 1. Fastest yearly 100 m times for each of the 10 fastest American male sprinters as a function of age. Graph of Top Ten Men Pooled plots the fastest yearly 100 m times for all sprinters pooled as a function of age. The curve for the best-fit quadratic equation ± 95% Confidence Intervals is indicated. Thirty-nine percent of the variation in fastest yearly 100 m time is explained by age.
